# YOLOv5s-Fog: An Improved Model Based on YOLOv5s for Object Detection in Foggy Weather Scenarios

**DOI:** 10.3390/s23115321

**Published:** 2023-06-03

**Authors:** Xianglin Meng, Yi Liu, Lili Fan, Jingjing Fan

**Affiliations:** 1 School of Electrical and Control Engineering, North China University of Technology, Beijing 100144, China; 2 National Industrial Innovation Center of Intelligent Equipment, Changzhou 213300, China; yliu@niicie.com (Y.L.); llfan18@mails.jlu.edu.cn (L.F.)

**Keywords:** foggy weather scenarios, deep learning, SwinFoucs, decoupled head, soft-NMS

## Abstract

In foggy weather scenarios, the scattering and absorption of light by water droplets and particulate matter cause object features in images to become blurred or lost, presenting a significant challenge for target detection in autonomous driving vehicles. To address this issue, this study proposes a foggy weather detection method based on the YOLOv5s framework, named YOLOv5s-Fog. The model enhances the feature extraction and expression capabilities of YOLOv5s by introducing a novel target detection layer called SwinFocus. Additionally, the decoupled head is incorporated into the model, and the conventional non-maximum suppression method is replaced with Soft-NMS. The experimental results demonstrate that these improvements effectively enhance the detection performance for blurry objects and small targets in foggy weather conditions. Compared to the baseline model, YOLOv5s, YOLOv5s-Fog achieves a 5.4% increase in mAP on the RTTS dataset, reaching 73.4%. This method provides technical support for rapid and accurate target detection in adverse weather conditions, such as foggy weather, for autonomous driving vehicles.

## 1. Introduction

In the field of autonomous driving, object detection is a crucial technology [1], and its accuracy and robustness are of paramount importance for practical applications [2]. However, in foggy weather scenarios, challenges arise due to weakened light and issues such as blurred object edges, which lead to a decline in algorithm performance, consequently affecting the safety and reliability of autonomous vehicles [3]. Therefore, conducting research on target detection in foggy weather scenes holds great significance.

In recent years, researchers have made certain progress in addressing the problem of object detection in foggy weather conditions [4,5]. Traditional methods primarily rely on conventional computer vision techniques such as edge detection, filtering, and background modeling. While these methods can partially handle foggy images, their effectiveness in complex scenes and under challenging foggy conditions is limited. To address the issue of object detection in complex foggy scenes, scholars have started exploring the utilization of physical models to represent foggy images. He et al. [6] proposed a single-image dehazing method based on the dark channel prior, while Zhu et al. [7] presented a fast single-image dehazing approach based on color attenuation prior. These dehazing methods improve the visibility of foggy images and subsequently enhance the accuracy of object detection. However, physical model-based methods require the estimation of fog density, making it difficult to handle multiple fog densities in complex scenes.

With the continuous development of deep learning techniques, deep learning has gradually become a research hotspot in the field of object detection [8,9]. Compared to traditional methods, deep learning models can directly learn tasks from raw data and exhibit improved generalization through training on large-scale datasets [10]. Deep learning-based object detection algorithms can be categorized into two-stage detectors and one-stage detectors. Two-stage detectors first generate a set of candidate boxes and then perform classification and position regression for each candidate box. Faster R-CNN [11] is the most representative algorithm in this category, which employs an RPN [11] to generate candidate boxes and utilizes ROI Pooling [12] for classification and position regression of each candidate box. In addressing the problem of object detection in foggy weather conditions, Chen et al. [13] proposed a domain adaptive method that aligns features and adapts domains between source and target domains, thereby improving the detection performance in the target domain. However, region proposal-based methods require more computational resources and incur higher costs, making them less suitable for real-time applications with stringent timing requirements [14].

One-stage detectors directly perform classification and position regression on the input image without the need for generating candidate boxes. The most representative algorithms in this category are the YOLO series [15,16,17] and SSD [18]. YOLO divides the image into a grid and predicts bounding boxes and class probabilities for each grid cell, while SSD predicts bounding boxes of different sizes on different feature layers. Compared to two-stage detectors, one-stage detectors have a significant advantage in terms of speed, making them particularly suitable for real-time applications. In previous studies, Qiu et al. [19] combined the coordinated attention (CA) [20] mechanism with the GhostNet [21] algorithm to improve the accuracy of the YOLOv5 algorithm, providing technical support for fast identification of multiple foxtail millet ear targets in complex field environments. Fan et al. [22] proposed a solution to reduce mispicking and missed picking of strawberry fruits by combining YOLOv5 with dark channel [6] enhancement. This study addressed the issue of low illumination during nighttime image capture and compared the detection results of five image enhancement algorithms, namely histogram equalization [23], Laplace transform [24], gamma transform [25], logarithmic variation, and dark channel [6] enhancement processing, across different time periods. The final results demonstrated that YOLOv5 outperformed SSD [18], DSSD [26], and EfficientDet [27] in terms of recognition accuracy, with a correct rate exceeding 90%. Baidya et al. [28] added an additional detection head to YOLOv5 and incorporated ConvMixers [29] in the context of unmanned aerial vehicle detection scenarios. They trained and tested the proposed architecture on the VisDrone2021 dataset, achieving results comparable to state-of-the-art methods. Ge et al. [30] embedded the Coordinated Attention [20] (CA) module and the Squeeze-and-Excitation [31] (SE) module into the YOLOv5s network for underwater target detection. The modified YOLOv5s showed a 2.4% improvement in mean average mAP compared to the baseline model.

However, improving the accuracy of object detection in complex weather and lighting conditions remains a challenge. To tackle this challenging problem, Huang et al. [32] employed two subnetworks to jointly learn visibility enhancement and object detection, reducing the impact of image degradation by sharing feature extraction layers. Hang et al. [33] and Guo et al. [34], on the other hand, addressed the issue by employing image defogging and image enhancement techniques, respectively, to mitigate the influence of weather-specific information. Hnewa et al. [35] proposed a cross-domain object detection method that utilizes multi-scale features and domain adaptation techniques to enhance the detection performance in complex weather conditions. Liu et al. [36] designed a fully differentiable image processing module based on YOLOv3 [15] for object detection in foggy and low-light scenarios. Although this image-adaptive approach improves detection accuracy, it also introduces some undesirable noise.

In the aforementioned research on foggy weather object detection, although the detection accuracy has been improved, most of these methods are primarily focused on defogging and image enhancement [37]. This study aims to enable object detection algorithms to achieve clear detection in foggy weather scenes without any preprocessing of the original image. In recent years, the application of Transformer models [38,39,40] in computer vision has been increasing. These models leverage self-attention mechanisms to capture relationships within an image, thereby enhancing model performance. In this study, the Swin Transformer [40] component is incorporated into the YOLOv5s model to improve detection accuracy in adverse weather conditions.

The main contributions of this study are as follows:

1. On the basis of the YOLOv5s model, we introduce a multi-scale attention feature detection layer called SwinFocus, based on the Swin Transformer, to better capture the correlations among different regions in foggy images;
2. The traditional YOLO Head is replaced with a decoupled head, which decomposes the object detection task into different subtasks, reducing the model’s reliance on specific regions in the input image;
3. In the stage of non-maximum suppression (NMS), Soft-NMS is employed to better preserve the target information, thereby effectively reducing issues such as false positives and false negatives.

The remaining sections of this paper are organized as follows. In Section 2, we provide a brief overview of the original YOLOv5s model and elaborate on the innovations proposed in this study. Section 3 presents the dataset, experimental details, and results obtained in our experiments. Finally, in Section 4, we summarize our work and propose some future research directions.

## 2. YOLOv5s-Fog

### 2.1. Overview of YOLOv5

**Figure 1:**
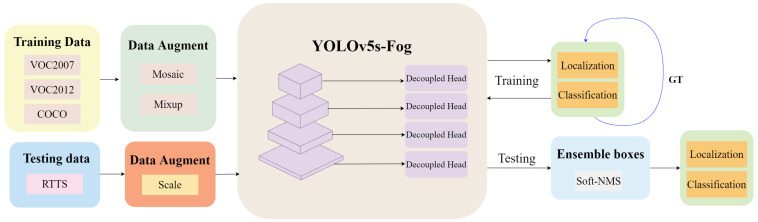
Operational procedure of YOLOv5s-Fog. This framework incorporates an augmented predictive feature layer to bolster the network’s regional comprehension. Additionally, we employ a decoupled head to effectively address scenarios characterized by diminished contrast and indistinct boundaries. Lastly, the Soft-NMS technique is employed for the integration of bounding boxes.

YOLOv5 [17] is an efficient and highly accurate real-time object detection algorithm that extends the YOLO series [15,16]. This algorithm employs a single neural network to perform bounding box and category predictions. In comparison to its previous versions, YOLOv5 incorporates several improvements, including a new backbone network based on the CSP architecture [41], dynamic anchor allocation methods, and data augmentation techniques such as Mixup [42]. These enhancements have enabled the algorithm to achieve outstanding performance on multiple benchmark datasets while maintaining real-time inference speeds on both CPU and GPU platforms. The YOLOv5 model consists of four different configurations: YOLOv5s, YOLOv5m, YOLOv5l, and YOLOv5x. In general, YOLOv5s is well-suited for real-time object detection in scenarios with limited computational resources, while YOLOv5x is more suitable for applications that require high-precision detection. Considering the real-time detection requirements in foggy weather conditions, this study employs YOLOv5s as the experimental model. The operational flow of YOLOv5s-Fog proposed in this paper is illustrated in Figure 1.

### 2.2. The Architecture of YOLOv5s-Fog Network

**Figure 2:**
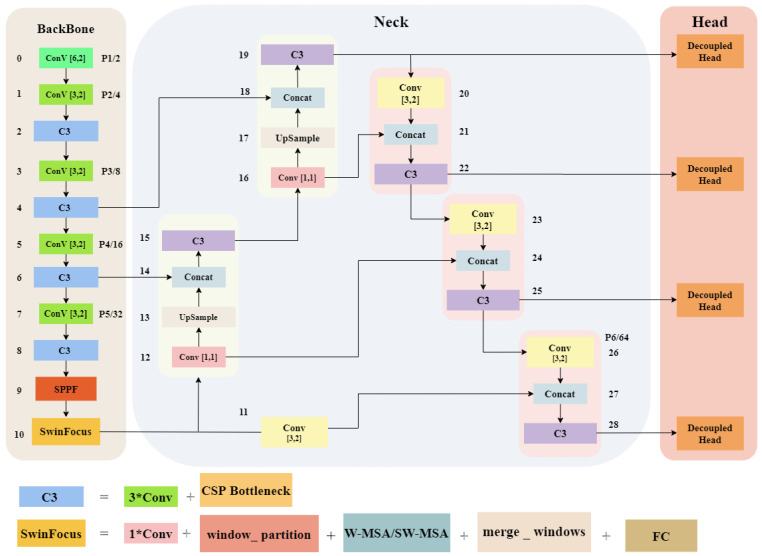
The network architecture of YOLOv5s-Fog introduces the following enhancements compared to the original version: addition of a target detection layer called SwinFocus based on Swin Transformer; use of a decoupled detection head to accomplish the final stage of the detection task. (The numbers 1–28 correspond to the numbering of each layer in the network architecture.)

In foggy weather conditions, object features in images often become blurry or even lost due to the presence of fog [43]. In this paper, we propose a novel approach to address the aforementioned issue by improving the YOLOv5s network architecture. Our proposed network, YOLOv5s-Fog, is illustrated in Figure 2. Firstly, we introduce a new feature detection layer called SwinFocus, which enhances the object detection capability by better capturing subtle features of objects in the image. Compared to traditional convolutional neural networks, SwinFocus achieves global interaction and aggregation of feature map information by decomposing the spatial and channel dimensions of the feature maps, enabling the network to better detect objects concealed in the fog. Secondly, to enhance the flexibility of the model during the detection stage, we employ a decoupled head, where the classification and regression heads are separately processed, making better use of the network’s expressive power. Finally, we utilize Soft-NMS in the post-processing stage to effectively handle the issue of overlapping objects in foggy images.

### 2.3. Construction of Object Detection Model for Foggy Scenes

#### 2.3.1. The Swin Transformer Architecture

**Figure 3:**
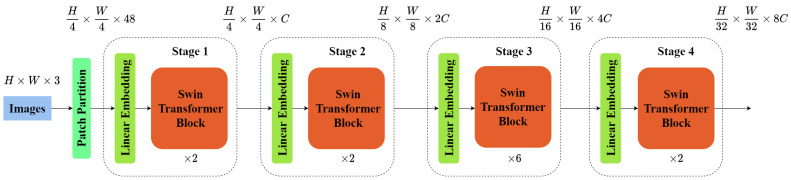
Swin Transformer Architecture.

In challenging weather conditions such as foggy environments, traditional Convolutional Neural Networks (CNNs) face a range of limitations and challenges in object detection tasks [44]. Firstly, the presence of fog causes image blurring, reduced contrast, and color distortion, making it difficult for traditional convolutional operations to effectively extract clear object edges and fine details. Secondly, lighting variations and occlusions in foggy scenes make it challenging for traditional CNNs to accurately localize and detect objects. Swin Transformer [40] is a neural network based on the Transformer [38] architecture that has demonstrated outstanding performance in computer vision tasks such as image classification, object detection, and semantic segmentation. The architecture of Swin Transformer is illustrated in Figure 3.

Unlike traditional CNNs, Swin Transformer introduces the Patch Partition module, which divides the input image into blocks and flattens them in the channel dimension to better capture the subtle features of objects in the image. In Swin Transformer, the image is first input into the Patch Partition module for block-wise processing, where each 4 × 4 adjacent pixels form a patch that is then flattened in the channel dimension. Subsequently, four stages are constructed to generate feature maps of different sizes. Stage 1 utilizes a linear embedding layer, while the remaining three stages employ Patch Merging for downsampling. Finally, these stages are stacked in a repeated manner.

**Figure 4:**
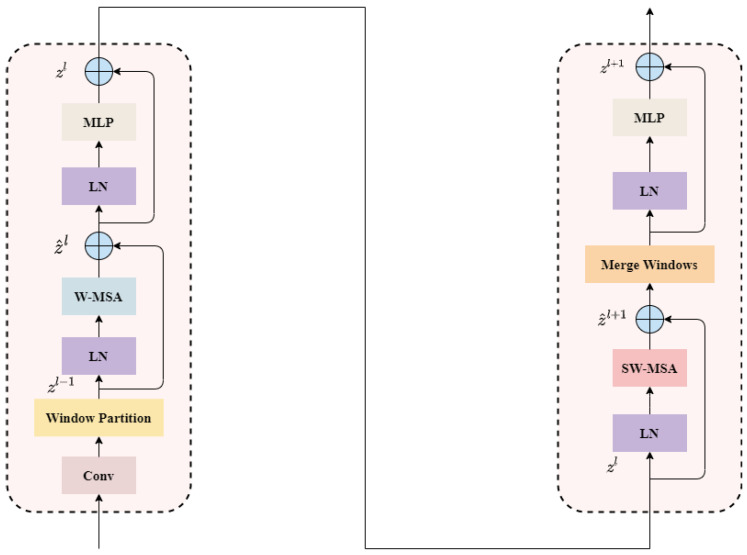
Two consecutive SwinFocus layers are employed in our approach. The SwinFocus layer introduces the concept of Windows Multi-head Self Attention (W-MSA), which significantly reduces computational complexity compared to the traditional Multi-head Self Attention (MSA). However, W-MSA performs self-attention calculations within each individual window. To enable information propagation between different windows, we further introduce the Shifted Windows Multi-Head Self-Attention (SW-MSA) mechanism.

To enhance the feature representation in adverse weather conditions, we introduce an additional Swin Transformer-based feature detection layer, SwinFocus, to the YOLOv5 framework. The basic structure of SwinFocus is illustrated in Figure 4. SwinFocus plays a critical role in object detection under challenging weather scenarios, with its hierarchical feature representation mechanism being the core component. Through multiple stages of downsampling, it can extract features at different scales, capturing information from objects of various sizes. This ability enables SwinFocus to adapt better to size variations and diversity in targets. Furthermore, SwinFocus inherits the window attention mechanism, which transforms global attention into local attention, allowing it to focus more on subtle details and edge information in the image. In foggy conditions where images may be affected by blurring and reduced visual quality, the window attention mechanism can precisely localize objects and extract crucial features. The computational formulas for two consecutive SwinFocus layers are as follows:

(1) $${\hat{z}}^{l}=W-MSA\left(LN\left(z^{l-1}\right)\right)+z^{l-1}$$

(2) $$z^{l}=MLP\left(LN\left({\hat{z}}^{l}\right)\right)+{\hat{z}}^{l}$$

(3) $${\hat{z}}^{l+1}=SW-MSA\left(LN\left(z^{l}\right)\right)+z^{l}$$

(4) $$z^{l+1}=MLP\left(LN\left({\hat{z}}^{l+1}\right)\right)+{\hat{z}}^{l+1}$$

#### 2.3.2. Decoupled Head

Deep learning-based object detection methods typically adopt a shared feature detector to simultaneously predict the class and location information of objects [45]. This coupling approach is beneficial as it improves model efficiency and accuracy through shared feature representations. However, in foggy weather conditions, this tightly coupled approach may face limitations and challenges. Firstly, the image quality in foggy environments is severely affected, resulting in visual impairments that make it difficult for traditional shared feature detectors to accurately extract clear object edges and fine details. Consequently, this impacts the accuracy of object localization and detection. Secondly, due to light absorption and scattering effects in foggy weather, the visibility of objects is reduced, causing indistinct object edges and easy blending with the background.

**Figure 5:**
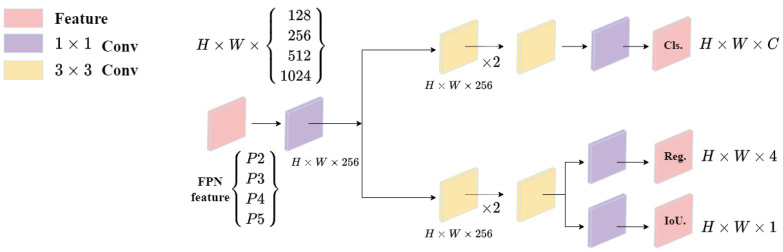
Decoupled Head Structure. Decoupled head is a multi-task learning approach that divides object detection into two steps: image classification and object localization within the image.(YOLOv5s-Fog incorporates four detection heads).

The Decoupled Head separates feature extraction from spatial position information by employing two independent network heads [46]. This design effectively reduces the coupling between feature extraction and spatial position information [45], enabling the model to better handle complex lighting variations caused by light propagation attenuation and scattering in foggy weather conditions. Moreover, the separation of feature extraction and spatial localization tasks allows the feature extraction head to focus on extracting discriminative features, while the spatial information head can concentrate on processing positional information. The structure of the decoupled head, as illustrated in Figure 5, involves a 1 × 1 convolutional layer to reduce the channel dimension, followed by two parallel branches, each containing two 3 × 3 convolutional layers [46]. This approach not only reduces the complexity of the network architecture but also enhances the accuracy of the model.

#### 2.3.3. Soft-NMS

**Figure 6:**
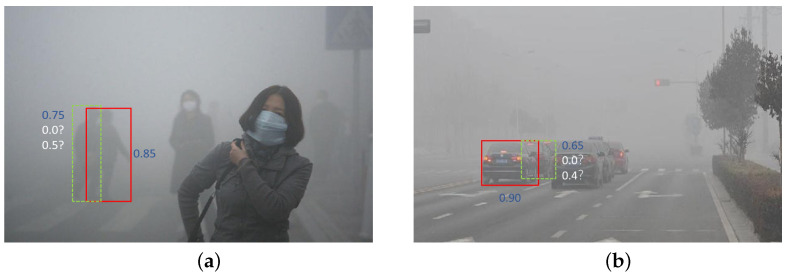
The issues that can occur during the post-processing stage of NMS. In (**a**), there are two reliable pedestrian detections (green bounding box and red bounding box) with scores of 0.85 and 0.75, respectively. However, due to the significant overlap between the green and red bounding boxes, the green bounding box is assigned a lower score. The situation in (**b**) is similar to that in (**a**).

In comparison to normal weather conditions, foggy weather exhibits differences in light propagation, contrast, color, and visibility [1]. These issues result in more prominent overlapping of objects. Traditional non-maximum suppression (NMS) methods may excessively suppress overlapping bounding boxes when selecting the one with the highest confidence, leading to the erroneous exclusion of important objects [47]. The specific details are shown in Figure 6. Soft-NMS addresses this by introducing a confidence decay factor, which helps preserve the confidence information of overlapping objects to a certain extent and reduces the likelihood of suppressing important objects. Additionally, traditional NMS methods solely rely on the intersection over union (IoU) between bounding boxes for suppression, disregarding the confidence information of the objects [48]. This can cause low-confidence bounding boxes to have a high IoU, while high-confidence bounding boxes may be erroneously suppressed. Soft-NMS adjusts the suppression based on both the confidence and overlap of the bounding boxes, thereby better preserving high-confidence bounding boxes and improving the localization accuracy of objects in foggy weather conditions.

Compared to traditional non-maximum suppression (NMS), Soft-NMS introduces a softening function that gradually reduces the scores of other bounding boxes overlapping with the one having the highest confidence, instead of directly setting their scores to zero [47]. This principle can be represented by the following formula: for a set of input bounding boxes $B=\left\{b_{1},b_{2},...,b_{n}\right\}$,where each bounding box $b_{i}$ consists of four coordinates and a confidence score $s_{i}$, Soft-NMS measures their similarity by computing the Intersection over Union (IoU) values between the boxes.

(5) $$IoU\left(b_{i},b_{j}\right)=\frac{b_{i}\cap b_{j}}{b_{i}\cup b_{j}}$$

Then, the scores of each detection box are adjusted based on their similarity. Specifically, for the currently processed detection box $b_{i}$, its final weight is given by:

(6) $${\omega_{i}}^{*}=\left\{\begin{array}{cc} s_{i} & \mathrm{if}\;s_{i}>\theta \\ e^{-\frac{{\left(IoU\left(b_{i},b_{k}\right)\right)}^{2}}{\sigma }}\cdot s_{k} & \mathrm{otherwise} \end{array}\right.$$

In this equation, θ represents a threshold. When $s_{i}$ is greater than θ, the original score is retained. Otherwise, a Gaussian function is used to suppress other similar detection boxes, with σ controlling the rate of weight reduction. The final weight ${\omega_{i}}^{*}$ is adjusted based on a linear interpolation with the confidence score $s_{i}$ of the current detection box.

(7) $$\omega_{i}=\left(1-\alpha \right)s_{i}+\alpha {\omega_{i}}^{*}$$

Among them, α is a parameter that controls the ratio between the adjusted score and the original score. Finally, for each detection box $b_{i}$, the Soft-NMS function adjusts it to $\omega_{i}$, where detection boxes with higher similarity will appear with lower weights in the output results, thus avoiding issues such as excessive suppression and exclusion of correct detections. Soft-NMS gradually reduces the scores of overlapping bounding boxes while preserving a certain degree of overlap. This approach allows for better handling of occlusions, blurriness, and overlapping instances in complex environments during object detection tasks. As a result, the selection of object detection boxes becomes more reasonable and stable, enhancing the overall performance of the detection system.

## 3. Experimental Setup and Results

### 3.1. Dataset

Insufficient datasets are available for training and testing object detection algorithms under adverse weather conditions, which can adversely affect their performance, particularly those based on a CNN. Additionally, the traditional atmospheric scattering model [15] fails to accurately simulate real-world foggy scenes [36]. To ensure fairness, we selected a total of 8201 images as the training set (V_C_t), sourced from VOC [49] and COCO [50] datasets. For the test set, we utilized V_n_ts [36] and RTTS [51]. RTTS was employed to evaluate the method’s object detection capability in foggy weather conditions, while V_n_ts was used to assess its performance on standard datasets. The dataset encompasses five categories: people, cars, buses, bicycles, and motorcycles. Further details regarding dataset usage are presented in Table 1.

### 3.2. Experimental Details

The experimental setup of YOLOv5s-Fog is shown in Table 2. During the training process of this study, we employed various effective data augmentation techniques, including MixUP [42] and Mosaic [16]. Additionally, we utilized a cosine learning rate scheduling strategy, setting the initial learning rate to $3\times 10^{-4}$, batch size to 16, and conducting 30 iterations.

### 3.3. Evaluation Metrics

This study evaluates the detection performance of the model using mean Average Precision (mAP). mAP is a metric commonly used to assess the performance of object detection algorithms. It represents the average area under the Precision-Recall curve, which provides a comprehensive evaluation of both the localization accuracy and recognition accuracy of the classifier. A higher mAP value indicates better detection performance of the model. The specific calculation method is as follows:

(8) $$P=\frac{TP}{TP+FP}$$

(9) $$R=\frac{TP}{TP+FN}$$

(10) $$AP=\sum_{n}\left(R_{n}-R_{n-1}\right)P_{n}$$

(11) $$mAP=\frac{1}{N}\sum_{i=1}^{N}AP_{i}$$

In this context, TP represents True Positive, FP represents False Positive, FN represents False Negative, $R_{n}$ denotes Recall, $P_{n}$ represents the maximum Precision at that Recall, and *N* indicates the number of classes.

### 3.4. Experimental Results

**Figure 7:**
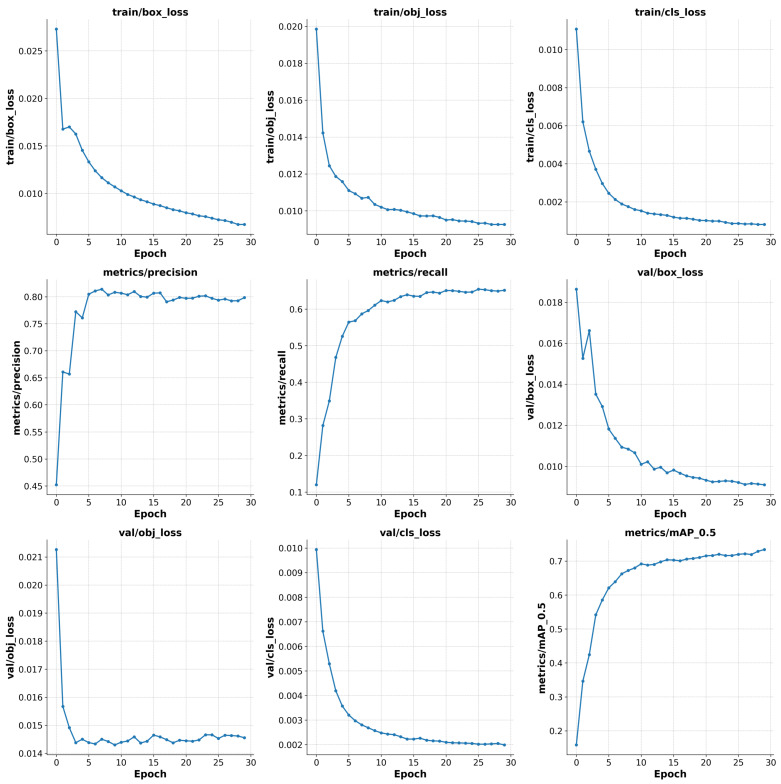
The visualization of various metrics during the training process, including bounding box loss, object loss, class loss, Precision, Recall, mAP, and mAP50-95.

To validate the effectiveness of YOLOv5s-Fog, we compared it with various existing methods for foggy scene object detection, including deep learning-based object detection networks [15,16,17], dehazing methods [33,52], domain adaptation [32,35], and image adaptive enhancement [36]. The specific results are shown in Table 3. Table 4 presents a comprehensive comparison between our proposed approach and state-of-the-art object detection models in terms of experimental results. Figure 7 illustrates the variations of key metrics, including bounding box loss, object loss, and class loss, as well as Precision, Recall, mAP, and mAP50-95 after each epoch during the training and validation process of YOLOv5s-Fog.

#### The Analysis of Experimental Results

**Figure 8:**
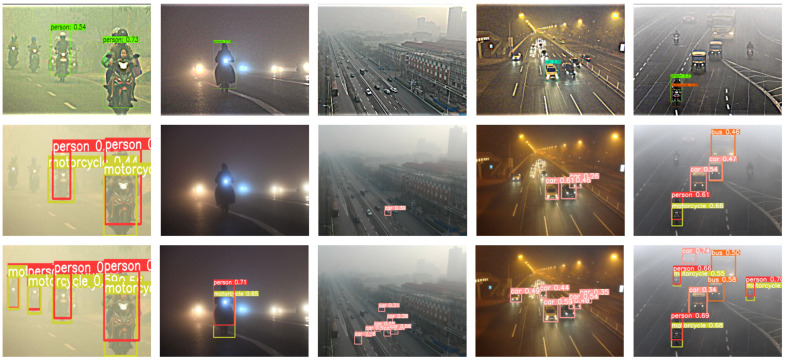
Partial detection results of IA-YOLO, YOLOv5s, and YOLOv5s-Fog on RTTS are shown below. The first row corresponds to IA-YOLO, the second row corresponds to YOLOv5s, and the third row corresponds to YOLOv5s-Fog.

From Table 3, it can be observed that YOLOv5s-Fog outperforms other methods both on conventional weather datasets and foggy weather datasets. Specifically, the combination of deep learning architecture with image-adaptive methods outperforms traditional image dehazing approaches. One notable example is IA-YOLO [36], which achieves performances of 72.65% and 36.73% on V_n_ts [49] and RTTS [51], respectively. This superiority can be attributed to the ability of image-adaptive algorithms to consider different regions within the image and make adaptive adjustments based on regional characteristics and requirements. In contrast, conventional dehazing [35] algorithms typically apply the same processing method to the entire image, without fully considering local variations within the image. IA-YOLO utilizes the YOLOv3 [15] network architecture. To further investigate the impact of network architecture on object detection results in foggy conditions, we conducted experiments using the original YOLOv5s [17]. YOLOv5s achieves significant improvements with performances of 87.56% on V_n_ts and 68% on RTTS compared to IA-YOLO. YOLOv5s incorporates a range of network architecture and techniques, including multi-scale fusion, anchor box design, and classifier optimization. Building upon YOLOv5s, YOLOv5s-Fog introduces additional feature detection layers [40] and a Decoupled Head [45,46] to enhance the network’s ability to explore challenging details in foggy scenes. Additionally, Soft-NMS [47] is employed in the post-processing stage to address occlusion issues in foggy conditions. Ultimately, our proposed method achieves mAP scores of 92.23% and 73.40% on V_n_ts and RTTS, respectively. Furthermore, YOLOv5s-Fog does not heavily focus on image dehazing, maintaining its original end-to-end detection approach and avoiding interference from artificially added noise during the detection phase. Figure 8 showcases partial detection results of the three models that performed well in RTTS. The first row presents IA-YOLO [36], which employs image adaptive techniques to remove specific weather information and restore the underlying content. Although this approach improves detection performance, it introduces undesired noise to the object detector. The second and third rows display the detection results of YOLOv5s and YOLOv5s-Fog, respectively, without image dehazing or image enhancement. It is evident from Figure 8 that YOLOv5s-Fog exhibits excellent detection capabilities in foggy weather conditions and low-light environments. Additionally, YOLOv5s-Fog can identify smaller objects in dense fog more effectively.

Table 4 clearly demonstrates that YOLOv5s-Fog outperforms YOLOv7 [54], both on the V_n_ts and RTTS datasets. The feature enhancement technique of YOLOv5s-Fog is specifically designed for foggy weather scenarios, and it achieves this with a smaller parameter count. In comparison to the YOLOv8 [56] series, YOLOv5s-Fog exhibits greater flexibility. While YOLOv8m and YOLOv8x may have superior performance, YOLOv5s-Fog offers significant advantages in terms of model complexity, as well as shorter training time.

### 3.5. Ablation Studies

In order to validate the effectiveness of each module, we conducted an ablation study on the RTTS dataset. To ensure the scientific rigor of this paper and comprehensively evaluate the proposed model, we employed three specific metrics: mAP, mAP50-95, and GFLOPs. The impact of each module on the detection results is listed in Table 5. Table 6 documents the detection performance of YOLOv5s-Fog on each object category in the RTTS dataset after incorporating different modules.

#### 3.5.1. The Impact of the Additional Feature Detection Layer

Through experimental validation, we have observed that SwinFocus significantly enhances the model’s mAP. This can be attributed to the adoption of the cross-domain self-attention mechanism during training, enabling the model to capture global features more effectively. Despite the introduction of additional object detection layers, which increase the model’s parameters and computational burden, it is justified considering the application scenarios in adverse weather conditions such as foggy weather. Table 5 demonstrates its notable performance improvements.

#### 3.5.2. The Impact of the Decoupled Head

By incorporating the Decoupled Head, the total number of layers in the model increased by 12, and the GFLOPs rose by 1.2. The adoption of the Decoupled Head not only enhances mAP but also enables adaptability to diverse object detection tasks and datasets, showcasing excellent scalability.

#### 3.5.3. The Impact of Soft-NMS

**Figure 9:**
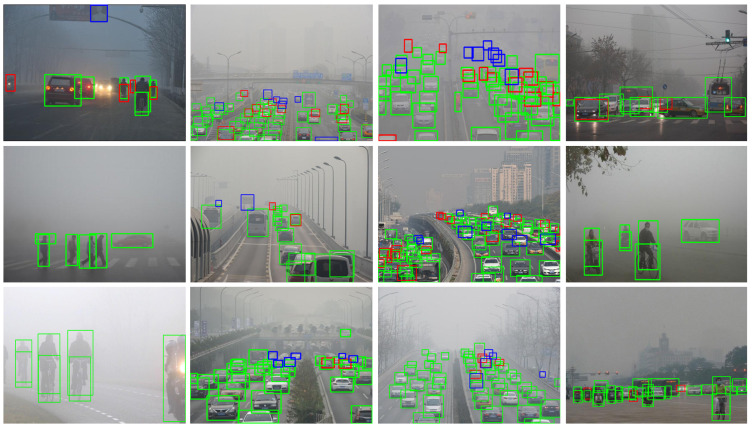
Visualization of the detection results of YOLOv5s-Fog on the RTTS dataset. The green, blue, and red boxes represent true positive (TP), false positive (FP), and false negative (FN) detections, respectively.

For object detection in foggy conditions, Soft-NMS primarily functions to address densely overlapping instances in large quantities. In Figure 9, we present the detection results of YOLOv5s-Fog on the RTTS dataset. Compared to traditional NMS, Soft-NMS exhibits superior handling of similar objects in complex environments, highlighting its significant advantage.

## 4. Conclusions

In this paper, we propose YOLOv5s-Fog, a novel approach to address the challenges of object detection under foggy conditions. Unlike previous research, we do not rely on dehazing or adaptive enhancement techniques applied to the original images. Instead, we enhance the YOLOv5s model by introducing additional detection layers and integrating advanced modules. Our improved model demonstrates higher accuracy in foggy conditions. Experimental results show the potential of our proposed method in object detection tasks under adverse weather conditions. In the future, we plan to invest more efforts in constructing datasets for object detection in extreme weather conditions and develop more efficient network architectures to enhance the model’s accuracy in extreme weather detection.

## Figures and Tables

**Table 1:** The relevant datasets used for training and testing purposes include V_C_t from VOC and COCO, V_n_ts from VOC2007_test, and RTTS, which is currently the only real-world foggy scene object detection dataset with multi-class detection labels.

Dataset	Image	Ps	Car	Bus	Bicycle	Motorcycle	Total
V_C_t	8201	14,012	3471	850	1478	1277	21,088
V_n_ts	2734	4528	337	1201	213	325	6604
RTTS	4322	7950	18,413	1838	534	862	29,597

**Table 2:** Experimental Setup of YOLOv5s-Fog.

Configuration	Parameter
CPU	Intel Xeon(R) CPU E5-2678 v3
GPU	Nvidia Titan Xp*2
Pytorch	1.12
CUDA	11.1
cuDNN	8.5.0

**Table 3:** Comparison of the performance of each method on the conventional dataset (V_n_ts) and the foggy weather dataset (RTTS). The rightmost two columns present the mAP(%) on the two test datasets, including V_n_ts and RTTS.

Methods	V_n_ts	RTTS
YOLOv3 [15]	64.13	28.82
YOLOv3-SPP [53]	70.10	30.80
YOLOv4 [16]	79.84	35.15
MSBDN [33]	/	30.20
GridDehaze [52]	/	32.41
DAYOLO [35]	56.51	29.93
DSNet [32]	53.29	28.91
IA-YOLO [36]	72.65	36.73
YOLOv5s [17]	87.56	68.00
YOLOv5s-Fog	92.23	73.40

**Table 4:** The experimental results present a comprehensive comparison between YOLOv5s-Fog and the state-of-the-art object detection algorithms, namely YOLOv7 and the YOLOv8 series. The “#Param.” is used to denote the number of model parameters. GFLOPs is a metric that measures the computational complexity or efficiency of the model. It represents the number of floating-point operations executed by the model per second, indicating the computational requirements.

Method	V_n_ts	RTTS	#Param.	GFLOPs
YOLOv7 [54]	92.83	72.16	36.9 M	104.70
YOLOv8n [55]	87.43	68.52	3.2 M	8.70
YOLOv8s	88.52	70.68	11.2 M	28.60
YOLOv8m	90.39	72.71	25.9 M	78.90
YOLOv8l	93.76	73.58	43.7 M	165.20
YOLOv8x	93.80	73.71	68.2 M	257.80
YOLOv5s-Fog	92.23	73.40	26.9 M	59.0

**Table 5:** The ablation experiment on the RTTS Dataset. (The green arrows and numbers indicate the specific improvement values of mAP after the addition of the module.)

Methods	mAP (%)	mAP50-95 (%)	GFLOPs
YOLOv5s	68.00	41.17	15.8
YOLOv5s + SwinFocus	70.15 (↑2.15)	43.40 (↑2.23)	56.2
YOLOv5s + SwinFocus + Decoupled Head	71.79 (↑1.64)	44.38 (↑0.98)	57.4
YOLOv5s + SwinFocus + Decoupled Head + Soft-NMS	73.40 (↑1.61)	45.58 (↑1.20)	59.0

**Table 6:** The impact of incorporating the component on the Precision (P) and Recall (R) of the model was evaluated on the RTTS dataset. Among the variants, YOLOv5s-Fog_1 represents the combination of YOLOv5s and SwinFocus. YOLOv5s-Fog_2 includes YOLOv5s, SwinFocus, and a Decoupled Head. Lastly, YOLOv5s-Fog_3 combines YOLOv5s, SwinFocus, a Decoupled Head, and Soft-NMS.

Methods	P						R					
	All	Person	Car	Bus	Bicycle	Motorcycle	All	Person	Car	Bus	Bicycle	Motorcycle
YOLOv5s	0.87	0.912	0.926	0.795	0.86	0.856	0.489	0.725	0.504	0.318	0.485	0.413
YOLOv5s-Fog_1	0.74	0.69	0.911	0.753	0.647	0.7	0.635	0.641	0.632	0.496	0.697	0.712
YOLOv5s-Fog_2	0.88	0.924	0.938	0.835	0.83	0.88	0.55	0.735	0.51	0.397	0.614	0.493
YOLOv5s-Fog_3	0.78	0.851	0.762	0.675	0.81	0.807	0.70	0.809	0.793	0.601	0.694	0.631

## Data Availability

The data presented in this study are available upon request from the corresponding author.
